# Single iterated fractal inspired UWB antenna with reconfigurable notch bands for compact electronics

**DOI:** 10.1016/j.heliyon.2023.e21419

**Published:** 2023-10-24

**Authors:** Musa Hussain, Tanvir Islam, Mohammed S. Alzaidi, Dalia H. Elkamchouchi, Fahad N. Alsunaydih, Fahd Alsaleem, Khaled Alhassoon

**Affiliations:** a Department of Electrical Engineering Bahria University Islamabad Campus, Pakistan; b Department of Electrical and Computer Engineering, University of Houston, Houston, TX, 77204, USA; c Department of Electrical Engineering, College of Engineering, Taif University, P.O. Box 11099, Taif, 21944, Saudi Arabia; d Department of Information Technology, College of Computer and Information Sciences, Princess Nourah bint Abdulrahman University, P.O. Box 84428, Riyadh, 11671, Saudi Arabia; e Department of Electrical Engineering, College of Engineering, Qassim University, Unaizah, 56452, Saudi Arabia

**Keywords:** Fractal stubs, Compact antenna, UWB, Reconfigurable notch-band, Compact electronics

## Abstract

A simple, compact, and low-profile antenna operating over ultrawideband with high gain is presented in this manuscript. The antenna has dimensions of W × L = 19 mm × 21 mm and is placed on the rear side of the FR-4 substrate material. The antenna contains simple geometry, inspired from a circular fractals, which consists of a circular patch with a CPW feedline. The circular patch is loaded with two fractals patches at both top end of the substrate and the rectangular stub is loaded at the lower side, to improve the antenna's bandwidth. The constructed antenna offers a wide band of 3–13.5 GHz. The antenna geometry also contains three semicircular slots, which are etched to generate the notch bands. Each slot is etched step by step, giving notch bands at 3.9 GHz, 5.2 GHz, and 8.1 GHz. In the final stage, two diodes are added to attain reconfiguration. The antenna offers moderate gain and high radiation efficiency. The hardware model of antenna is engineered to verify the simulated results. Moreover, the antenna is compared with other works in literature. The outcomes of the proposed antenna and comparison with the literature work make the suggested work the best candidate for future UWB portable devices.

## 1 Introduction

5G and 6G communication systems offer high data rates and low latency operations to manage many users simultaneously [1]. To meet these demands, the designing and manufacturing processes were revised, which also revised the design concept of components used in these communication models. As the antenna is a key part of communication technologies, these revisions and changes also affect antenna design requirements [2,3]. Future communication models require compact, wideband, and high-gain antennas with low manufacturing costs and weight [4]. For this purpose, the UWB antenna is considered to be the best because it is wideband and also has the advantages of low complexity, low profile, and low spatial power density [5,6].

With the advancement of technology, several sub bands have been introduced that lie inside the UWB spectrum. The existence of these bands may cause interference, which is eliminated by a notch-band antenna [7,8]. The notch-band antenna has the functionality to eliminate unwanted bands from the UWB region. This elimination of unwanted bands can be done by using band stop filters, which may increase the complexity and cost of the model and enhance its overall size [9]. Besides these setbacks, the presence of a filter with an antenna also causes mismatching and may increase losses. It looks very fruitful to make antenna systems capable of notching characteristics rather than introducing filters [10,11].

In the literature, the researcher adopts many techniques to get notch band characteristics, such as stub loading [12], slot insertion [13], loading metamaterials, or using defective ground structures [14]. In recent research, a notch-band antenna introduced the additional characteristic of reconfiguration [15]. Due to this characteristic, frequency band switching is performed [16]. After this technique, the selected frequency can be filtered and unfiltered as per system requirements [[17], [18], [19]]. In this literature review, some recent work is studied operating over UWB with only notch band characteristics [[20], [21], [22], [23], [24]] and notch band characteristics along with reconfiguration [[25], [26], [27], [28], [29], [30], [31]].

[20] presents a dual notch-band UWB antenna operating at 2.7–14.6 GHz with notch bands of 3.4–3.7 GHz and 5.1–5.8 GHz. The antenna's overall measurements are 48 mm × 24 mm × 1.2 mm, and its maximum gain is 2 dBi. The antenna offers a large frequency range and a notch band; however, it has poor gain and no reconfigurability [21]. describes a small notch-band antenna with overall dimensions of 24 mm × 30 mm × 1.2 mm. The antenna has a complex geometry and doesn't offer reconfigurability. Another compact antenna is given in Ref. [22], which has a compact size of 27 × 32 × 1 mm^3^, operates over an ultra-wide band of 1.8 dBi. The antenna has a low gain setback, and this work also does not offer switching.

As specified in Refs. [23,24], a UWB antenna provides a wideband of 3–12 GHz and 3–11 GHz, respectively. The antenna additionally offers a tri-notch band with a fair gain value of >4.5 dBi. Both the antennas in Refs. [23,24] include notch bands at 3.22–3.83 GHz, 4.49–5 GHz, and 7.49–8.02 GHz. The antenna in Ref. [23] also includes notch bands at 3–4.17 GHz, 5.33–6.5 GHz, and 8.5–12 GHz. Both works suffer from bigger dimensions and a lack of reconfiguration [23,24]. both have an overall dimension of 40 mm 28 mm 0.9 mm and 40 mm 29 mm 1.6 mm, respectively.

A notch-band antenna with reconfigurability shows more potential than an antenna with only notch-band characteristics. The PIN diodes, resisters, and varactor diodes are used for switching. The UWB design given in Ref. [25] operates at 1.4–11.3 GHz and gains 4.4 dBi. The antenna offers reconfigurability of notch bands along the triple bands of 1.8–2.3 GHz, 3.2–3.8 GHz, and 5.6–6.1 GHz. The antenna provides three controllable notches and has a simple structure but large dimensions. Another wideband design offering tri-notches is given in Ref. [26]. The antenna has the capability of reconfiguration by using PIN diodes but exhibits a large size of 50 mm × 60 mm. A small-sized antenna (20 mm × 20 mm) is presented in Ref. [27]. The antenna provides moderate gain of 4.25 dBi through UWB of 3.12–12 GHz. The merit of this work is its complex structure and dual notch at the ultra-wide frequency region.

In [28], a UWB antenna is presented with dual reconfigurable notches at 3.2–3.6 GHz and 4.32–5.81 GHz. The antenna has measurements of 37.8 mm × 27.1 mm × 1.6 mm with the highest value of gain around 3 dBi. The reconfiguration of notch bands is performed by using a double SRR structure. The antenna has a big size and a complex structure. Another dual-band but small-size antenna with dimensions of 24 mm × 24 mm is presented in Ref. [29]. The antenna offers a reconfigurable notch band between 2.8–4 GHz and 4.8–6.2 GHz using varactor diodes. The antenna also consists of an EBG structure, due to which the structure becomes complex. In Ref. [30], a simplified structure antenna is given, which provides a reconfigurable notch band on only one frequency band. The antenna has a huge size of 38.5 mm × 24 mm but offers a high value of gain of around 8.36. Another high-gain antenna (8 dBi) is reported for UWB applications [31]. The antenna offers notch reconfiguration around 3.43–3.75 GHz and 4.87–6.4 GHz. The complex structural arrangements are the major setback of this work, which is due to the SRR and its large size of 42 mm × 32 mm.

The above review helps in concluding this fact: there is still a research gap in designing UWB antennas with notch characteristics. The antenna either has limitations in terms of reconfigurable characteristics or is large. Some antennas also have a narrow notch band and low gain. This manuscript recommends an ultrawideband antenna with small and straightforward geometry, operating at high gain with a notch and reconfigurable characteristics. The contribution of this work is:

• Small and simplified Structure.
• The triple reconfigurable notch which covers full targeted band.
• Provides reconfigurability over UWB.

In the rest of the manuscript, the designing methodology and working principles are given in the second section. In the third section, the manicured prototype and its results are compared with the predicted results. In the final section, the work is concluded with a comparison table that compares suggested work with work in literature.

## 2 Design methodology of proposed antenna

### 2.1 Antenna design

**Fig. 1:**
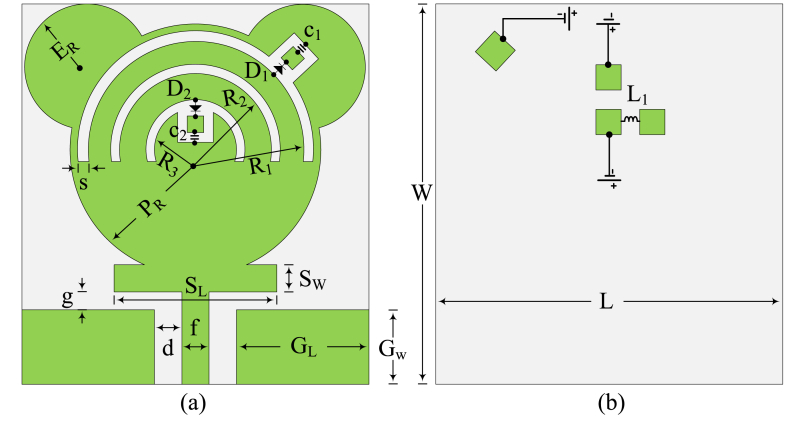
Geometrical illustration of presented reconfigurable notch band antenna (a) topside (b) bottom side.

Fig. 1 expresses the structure and geometry of the suggested antenna. It is notable from the figure that the antenna structure contains a circular patch with a co-planner waveguide (CPW) microstrip feed line. Circular stubs are loaded at the top edges, while rectangular stubs are placed between the circular patch and feedline. Moreover, the structure also contains three slots, where the *P*–I–N diodes are placed. To enhance the capabilities of the antenna, slots have been made, and these stubs are loaded. W × L = 19 mm × 21 mm is the total size of the antenna. On the top side of the readily available commercial substrate material FR–4, the suggested antenna is developed. The relative permittivity and loss tangent of the utilized substrate are 4.4 and 0.02, respectively. The thickness of the substrate material is 1.6 mm. The optimized parameters are given below:

P_R_ = 7; R_1_ = 6; R_2_ = 4.25; R_3_ = 2.25; E_R_ = 3.5; d = 0.5; f = 2; g = 1; s = 0.5; L = 19; W = 21; G_L_ = 8; G_W_ = 4; S_L_ = 9; S_W_ = 1.5. (Units are in mm)

### 2.2 Design of UWB antenna

**Fig. 2:**
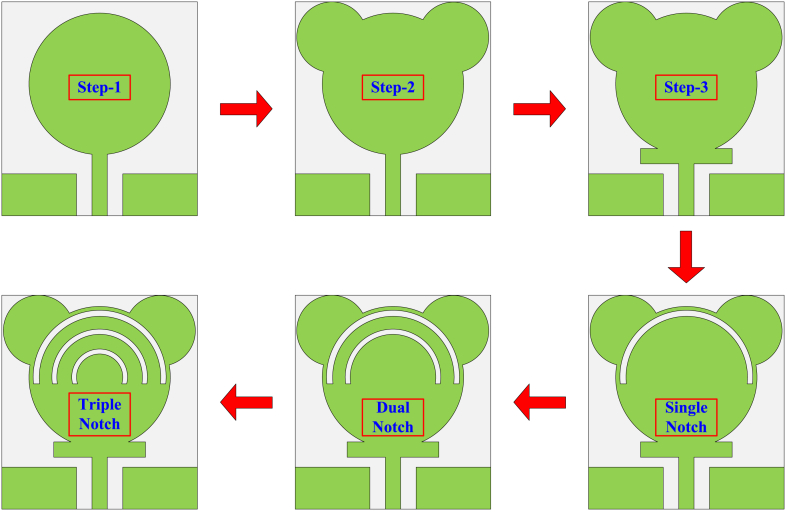
Construction stages of presented antenna with UWB and reconfigurable notch band antenna.

**Fig. 3:**
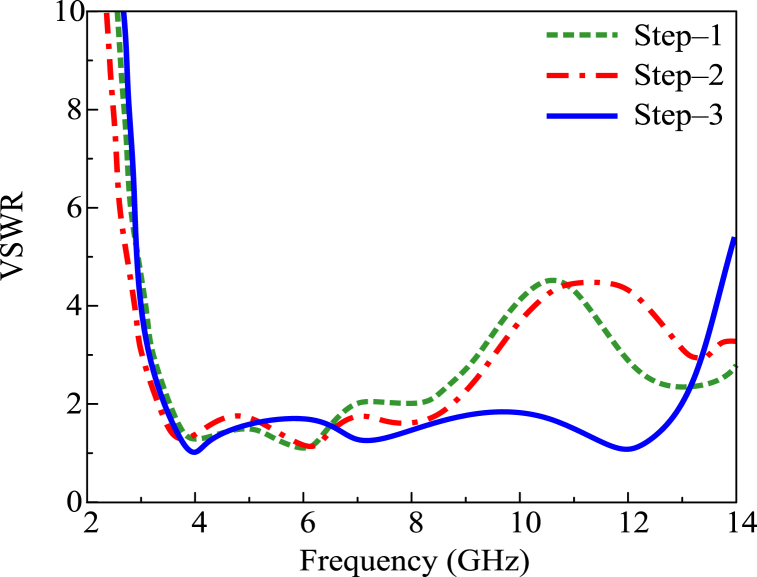
VSWR analysis of design stages while designing UWB antenna.

The antenna given in this paper was designed after following some design steps. In the first step, the circular patch antenna is designed with a radius P_R_ of 7 mm, which is obtained from the equations given in Ref. [32]. The antenna constructed by the initial step operates at 3.2–6.5 GHz, as given in Fig. 3. Afterward, two circular stubs having a radius of E_R_ = 3.5 mm are placed at the top of the circular patch in such a way as to meet the edges of the substrate, as given in Fig. 2. This stage shows an impact in terms of improvement in bandwidth. After the second stage, the impedance bandwidth is 3.2–9 GHz (VSRW <2). A stub is placed between the principal circular patch and the feedline in the third stage. The stub has length S_L_ = 9 mm and width S_W_ = 1.5 mm. After this stage of design, the antenna starts operating over the ultrawideband of 3.2–13.35 GHz with a VSWR value of less than 2.

The initial design stages of the UWB antenna are given in Fig. 2, whereas their impact in terms of VSWR is provided in Fig. 3.

### 2.3 UWB antenna with notch bands

**Fig. 4:**
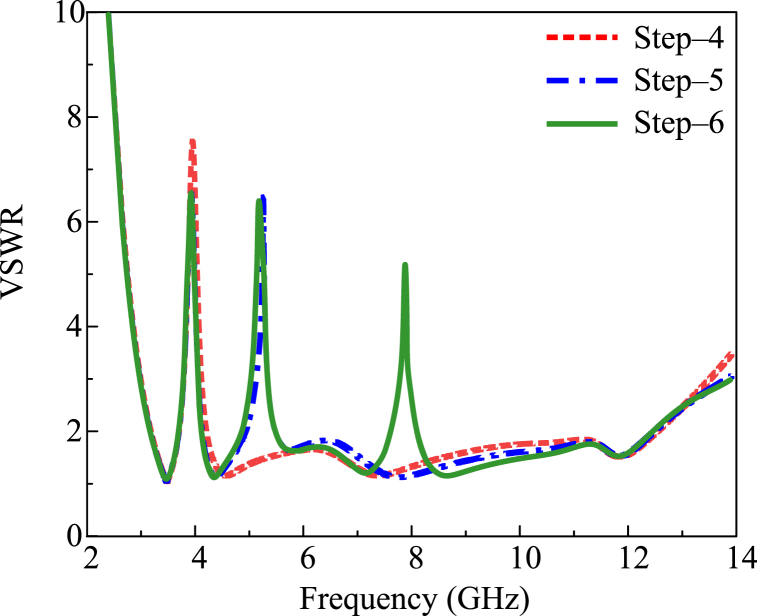
VSWR results with single, double, and tri-notch bands.

In order to get a notch band, some modifications are performed over the UWB antenna discussed in the previous section. In order to get the single notch band at 3.9 GHz with a bandwidth of 3.8–4.2 GHz, the semicircular arc is etched with a radius of R1 = 6 mm and a width of s = 0.5 mm, as given in Fig. 2. After loading the second semicircular arc with a radius of R2 = 4.25 mm, the dual notch band is observed. The second notch band is noticed at 5.1 GHz, having a bandwidth of 4.9–5.6 GHz, as given in Fig. 4. When the third semicircular slot having a radius of R3 = 2.25 mm is etched, the tri-notch band is observed as given in Fig. 4. The third notch band can be observed at 8 GHz, having a bandwidth of 7.8–8.2 GHz. The design stages to obtain the notch bands are given in Fig. 2, while their impact on the VSWR plot is given in Fig. 4.

### 2.4 Reconfigurable notch band antenna

In order to get reconfiguration between the notch bands, two PIN diodes are loaded into the antenna. One PIN diode is placed at the left top end between the first semicircular slot and circular patch, while the other is inserted between the second and third semicircular slots. The placement of the diode is finalized for on-demand notch band. The reconfigurability is achieved due to the radiator's electrical length and ground plane being changed to allow the current flow by switching diodes to the ON and OFF stages [33]. This leads to the addition of the PIN diode and the use of resistance-inductor-capacitor (RLC) components in the antenna design. A Dc block 100 pF capacitor is used to change the current flow path, and the RLC values are provided in [34]. In addition, padding and visas are included in the design framework for improved outcomes.

**Fig. 5:**
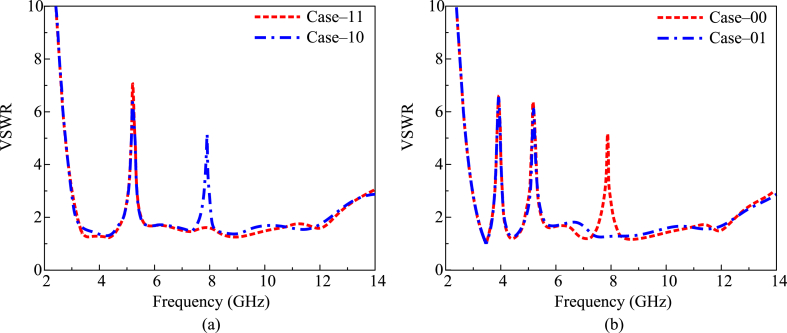
VSWR results with different states (a) case-11 & case-00 (b) case-00 & case-01.

After loading the PIN diode, the antenna provides a notch at 5 GHz when both diodes are ON (case 11). When diode C_1_ is ON and diode C_2_ is OFF, the antenna gives dual notches at 5 GHz and 8 GHz (case 10). In the third case, when diode C_1_ is in the OFF state and diode C_2_ is in the ON state, the antenna again gives dual notches at 3.9 GHz and 5 GHz (case 01). In the final case, when both diodes are in the OFF state, the antenna offers tri-notch bands at 3.9 GHz, 5 GHz, and 8 GHz, as given in Fig. 5 (case 00).

## 3 Results and discussion

This section discusses the tri-notch band antenna's outcomes in terms of reflection coefficient, gain, efficiency, surface current, and radiation pattern. This part includes explanations of the facts regarding the measuring setup and the fabrication of the prototype. The table of comparisons is provided at the end of the section to express how the suggested work is superior to the work that has already been presented.

### 3.1 Fabricated prototype

An electromagnetic (EM) software tool called High Frequency Structure Simulator (HFSS) is utilized to construct and analyze the suggested antenna geometry. Where the design of antennas, simulation of results, and design optimization are performed. The major advantage of using this software tool is that it minimizes the number of tests and verifications needed and the cost of the model, as it avoids fabricating many antennas for testing. The software above has various methods to work with complex geometries, including the finite element method (FEM) and the method of moments (MOM).

**Fig. 6:**
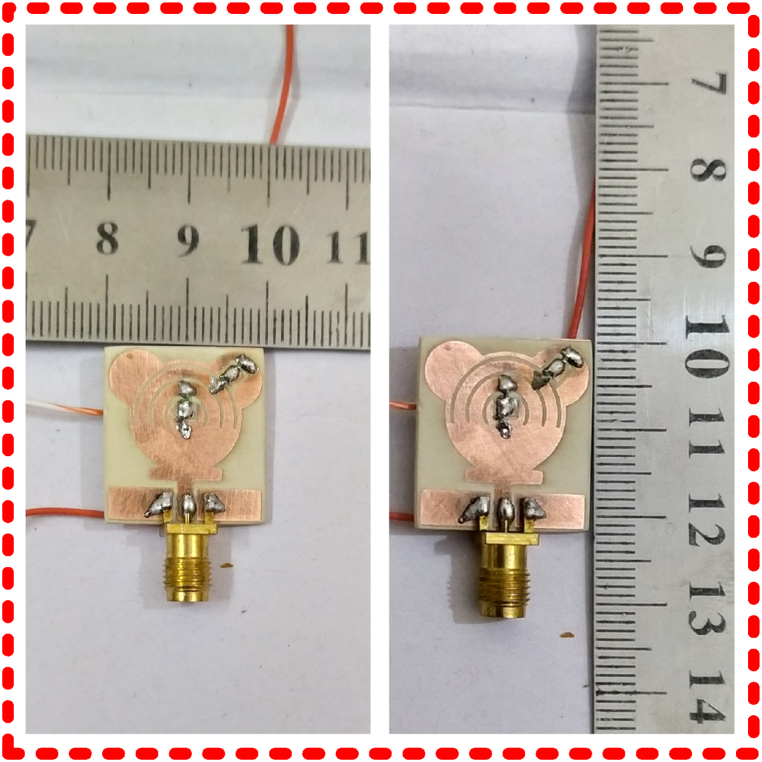
Fabricated prototype along with biasing circuitry utilized for measurement of various results.

Later on, a commercially available substrate material, FR-4, is utilized to fabricate the hardware prototype of the recommended work. The fabrication is performed to verify and validate the software-generated results. The Agilent vector network testing equipment is used to test the impedance and radiation pattern. The standard horn antenna is placed as a source antenna for the far-field measurements, whereas the suggested antenna is used as a receiving antenna. Fig. 6 shows the fabricated prototype of the suggested antenna. It can be observed from the figure that the antenna is fed by an SMA connector and loaded with a diode to provide reconfigurability. The compactness of the antenna can be seen by comparing it with the ruler scale.

### 3.2 VSWR comparison

**Fig. 7:**
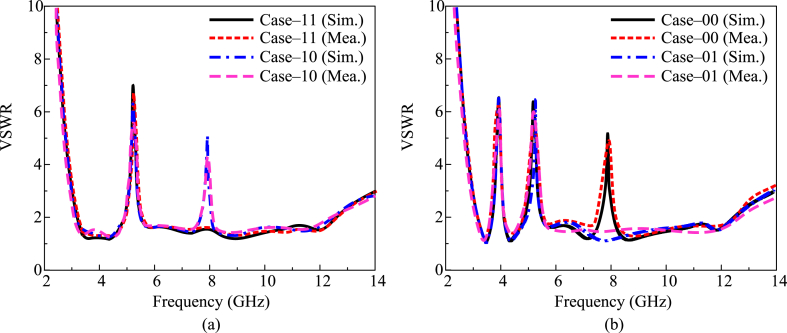
Comparison among predicated and hardware tested VSWR for (a) case-11 & case-10 (b) case-00 & case-01.

In Fig. 7, four scenarios of software-generated and prototype-tested VSWR are compared. When both diodes are turned on, it can be seen that the antenna offers a single notch at 5 GHz with a bandwidth of 4.9–5.8 GHz. As shown in Fig. 7(a), the antenna produces twin notches at 5 GHz and 8 GHz with bandwidths of 4.9–5.8 GHz and 7.6–8.2 GHz, respectively, when diode C1 is ON and diode C2 is OFF. In the third scenario, the antenna again produces twin notches at 3.9 GHz with a bandwidth of 3.7–4.2 GHz and 5.2 GHz with a bandwidth of 4.85–5.9 GHz while diode C1 is in the OFF state and diode C2 is in the ON state. The antenna offers tri-notch bands at 3.9 GHz, 5.2 GHz, and 8 GHz with bandwidths of 3.7–4.2 GHz, 4.8–5.9 GHz, and 7.6–8.3 GHz, respectively, in the final scenario when both diodes are in the OFF state, as shown in Fig. 7(b). Additionally, it is evident from the presented figure that the test and simulation findings for the antenna are pretty comparable to one another. The recommended work could be a candidate for the next UWB portable devices as a result of these findings.

### 3.3 Radiation pattern

**Fig. 8:**
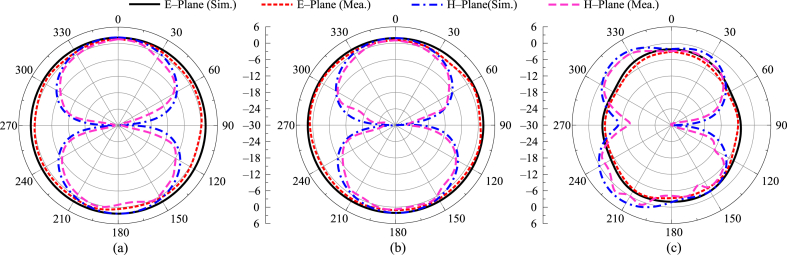
Comparison among radiation pattern for case-00 (a) 3.5 GHz (b) 4.4 GHz (c) 8.8 GHz.

The suggested tri-notch band antenna's simulated and tested radiation pattern at specific frequencies of 3.5 GHz, 4.4 GHz, and 8.8 GHz is shown in Fig. 8. The provided figures show that, at 3.5 GHz and 4.4 GHz, the antenna delivers a bidirectional radiation pattern in the E-plane and an omnidirectional radiation pattern in the H-plane, as shown in Fig. 8(a and b). The radiation pattern is slightly distorted for both the E and H planes at the high frequency of 8.8 GHz, as seen in Fig. 8(c). Additionally, the results from simulation and testing are identical.

### 3.4 Surface current analysis

**Fig. 9:**
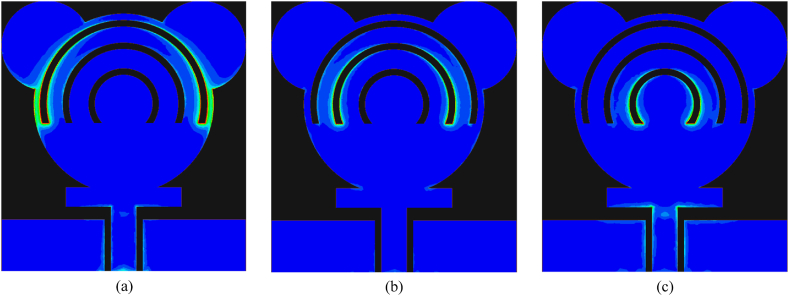
Surface current distribution of presented work for case-00 (a) 3.7 GHz (b) 5.2 GHz (c) 8 GHz.

The surface current of a tri-notch band antenna at selective frequencies of 3.7 GHz, 5.2 GHz, and 8 GHz is illustrated in Fig. 9(a–c). It is the key performance parameter for studying the notch-band antenna. At 3.7 GHz, it is notable that current is mostly focused on the edges of the topmost semicircular slot, while for the second resonant frequency, surface current is observed at the second semicircular slot. Similarly, for the third resonance, most of the surface current is seen at the third semicircular slot and slightly at the edges of the rectangular stub and feedline. These results show that the first notch band is provided by the initial semicircular slot, and the second notch is due to the mid-semicircular slot. The third semicircular slot generates the third resonance. These surface current distribution plots prove the theory of designing a notch-band antenna given in Section 2.3.

### 3.5 Gain of the proposed antenna

**Fig. 10:**
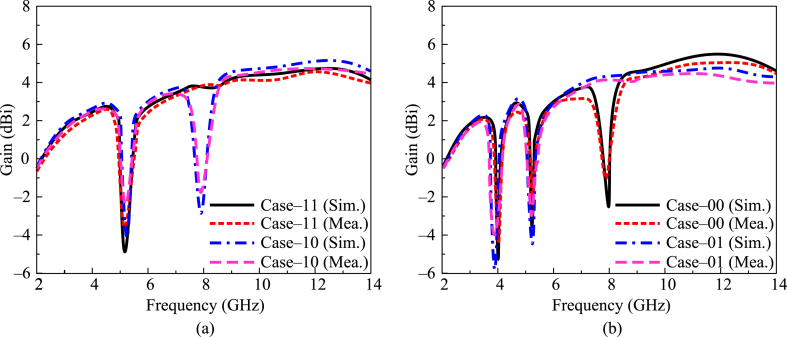
Gain versus frequency plot of presented work (a) case 11 & case 10 (b) case 00 & case 01.

The gain versus frequency plot of the suggested antenna for all possible states of diode switching is given in Fig. 10. It can be observed that antennas provide a gain of more than 3 dBi in the operational bandwidth of 3.2–13.5 GHz. By switching both diodes ON, the antenna operates in a.

Single notch band and offers gain greater than 3 dBi at operational bandwidth, whereas gain is less than −4 dBi at the notch band region. In case 10, when one diode is ON while the other is OFF, the antenna offers a dual-notch band. The antenna offers gain >3.25 dBi through the operational region. The value of gain decreased to < −2.5 dBi at notch band regions, as given in Fig. 10(a). In the third case, when C_1_ is OFF and diode C_2_ is ON, the antenna offers dual notch bands. The antenna under this scenario offers gain >2.75 dBi at operational bandwidth, while the gain goes to negative at notch band regions. In the final case, when both of the diodes are OFF, the antenna offers three notch bands. At this antenna, gain is > 3 dBi, while at the notch region, antenna gain decreases, as given in Fig. 10 (b).

### 3.6 Radiation efficiency

**Fig. 11:**
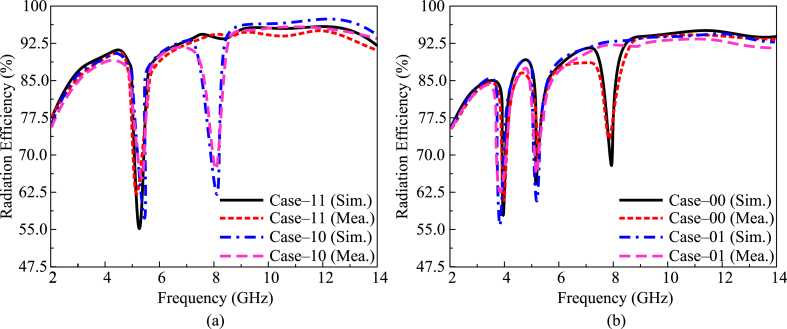
Radiation Efficiency of presented work (a) case 11 & case 10 (b) case 00 & case 01.

The radiation efficiency of the suggested tri-notch and UWB antenna at various switching states is given in Fig. 11(a and b). It can be observed that antennas provide a radiation efficiency greater than 90 % in the operational bandwidth of 3.2–13.5 GHz. By switching both diodes ON, the antenna operates in a single notch band and offers radiation efficiency greater than 88 % at operational bandwidth, whereas radiation efficiency is less than 50 % at the notch band region. In case 10, when one diode is ON while the other is OFF, the antenna offers a dual-notch band. The antenna offers radiation efficiency >89 % through the operational region. The value of radiation efficiency decreased to <50 % at notch band regions, as given in Fig. 11(a). In the third case, when C_1_ is OFF and diode C_2_ is ON, the antenna offers dual notch bands. The antenna under this scenario offers radiation efficiency greater than 85 % at operational bandwidth, while the gain goes to 60 % at notch band regions. In the final case, when both of the diodes are OFF, the antenna offers three notch bands. At this antenna, radiation efficiency is >88 %, while at the notch region, antenna radiation efficiency decreases, as given in Fig. 11(b).

### 3.7 Comparison with literature work

Table 1 compares the proposed work to antenna designs that have already been published in the state of the art. The table makes it evident that the suggested tri-notch band and UWB antenna are small in size and operate over a large frequency range of 3–13.5 GHz. The antenna offers moderate value with a triple-notch band. The antenna also provides on-demand reconfigurability between notch bands. This comparison makes the suggested work a potential candidate for future portable devices.

**Table 1:** Comparison with Recently reported UWB antennas.

Ref	Size (mm3)	Bandwidth (GHz)	Peak Gain (dBi)	Notch Band Ranges (GHz)	No. Notch Band	Reconfiguration
[20]	48 × 24 × 1.2	2.7–14.6	2	3.4–3.7 | 5.1–5.8	2	No
[21]	24 × 30 × 1.2	2.86–12.2	4.3	3.25–3.5 | 3.7–4.2 | 5.2–5.9 | 7–7.8	4	No
[22]	27 × 32 × 1	2.8–10	1.8	3.06–3.83 | 5–5.96	2	No
[23]	40 × 28 × 0.9	3–12	4.9	3–4.17 | 5.33–6.5 | 8.5–12	3	No
[24]	40 × 29 × 1.6	3–11	4.5	3.22–3.83 | 4.49–5 | 7.49–8.02	3	No
[25]	33 × 34 × 1.6	1.4–11.3	4.4	1.8–2.3 | 3.2–3.8 |5.6–6.1	3	Yes
[26]	50 × 60 × 1	2–20	–	2.77–3.3 | 5.51–6.25	2	Yes
[27]	20 × 20 × 0.8	3.12–12	4.25	3.12–3.82 | 4.9–6	2	Yes
[28]	37.8 × 27.1 × 1.6	3.1–11.6	3	3.2–3.67 | 4.32–5.81	2	Yes
[29]	24 × 24 × 1.6	3–12	–	2.8–4 | 4.7–6.2	2	Yes
[30]	38.5 × 24 × 1.6	3–12	8.36	3.89–5.03	1	Yes
[31]	42 × 32 × 1.6	3–9	8	3.43–3.75 | 4.87–6.4	2	Yes
This Work	19 × 21 × 1.6	3–13.5	4	3.7–4.2 | 4.8–5.9 | 7.6–8.3	3	Yes

## 4 Conclusions

In this work, an antenna is designed and investigated for three reconfigurable notch bands. The antenna has a compact size of 19 mm × 21 mm and operates over an ultrawide band of 3–13.5 GHz. In the first step, an UWB antenna is designed by loading stubs onto a circular patch antenna. Afterward, the semicircular slots are etched to get the notch bands. Three semicircular slots of different radiuses generate the notch bands of 3.7–4.2 GHz, 4.8–5.9 GHz, and 7.6–8.3 GHz. In the last stage, the PIN diodes are loaded for reconfiguration. The placement of two diodes gives four different cases of reconfiguration. In case 1, both of the diodes are in the ON state, and the antenna gives a single notch. The antenna produces two notch bands in the second and third situations when diode C1 is in the ON state and diode C2 is in the OFF state, and vice versa. In the fourth step, the antenna produces triple notch bands when both diodes are off. Additionally, the antenna has a high radiation efficiency of over 90 % and a moderate gain of about 4 dBi at operational bandwidth. Additionally, a hardware prototype is created to test the outcomes of the simulation. The suggested work is contrasted with previous research in the final section in terms of size, bandwidth, gain, and notch band. Additionally, the outcomes and comparison table demonstrate that the suggested antenna is the top contender for the next UWB portable devices.

## Funding

This work was supported by 10.13039/501100004242Princess Nourah bint Abdulrahman University Researchers Supporting Project number (PNURSP2023R238), 10.13039/501100004242Princess Nourah bint Abdulrahman University, Riyadh, Saudi Arabia.

## Data availability statement

Data included in article/supplementary material/referenced in article.

## CRediT authorship contribution statement

**Musa Hussain:** Writing – review & editing, Writing – original draft, Validation, Software, Methodology, Formal analysis, Data curation, Conceptualization. **Tanvir Islam:** Writing – original draft, Resources, Methodology, Data curation. **Mohammed S. Alzaidi:** Writing – original draft, Validation, Investigation, Funding acquisition, Formal analysis, Data curation. **Dalia H. Elkamchouchi:** Writing – original draft, Visualization, Resources, Conceptualization. **Fahad N. Alsunaydih:** Writing – review & editing, Writing – original draft, Visualization, Validation, Project administration, Funding acquisition. **Fahd Alsaleem:** Writing – review & editing, Writing – original draft, Visualization, Supervision, Methodology, Funding acquisition, Conceptualization. **Khaled Alhassoon:** Writing – review & editing, Writing – original draft, Visualization, Validation, Supervision, Resources, Project administration, Methodology, Investigation.

## Declaration of competing interest

The authors declare that they have no known competing financial interests or personal relationships that could have appeared to influence the work reported in this paper.

## References

[1] Hussain M., Awan W.A., Alzaidi M.S., Hussain N., Ali E.M., Falcone F. (2023). Metamaterials and their application in the performance enhancement of reconfigurable antennas: a review. Micromachines.

[2] Jarchavi S.M.R., Hussain M., Gardezi S.H.H., Alibakhshikenari M., Falcone F., Limiti E. (2022).

[3] Awan Wahaj A., Zaidi Abir, Hussain Niamat, Iqbal Amjad, Baghdad Abdennaceur (2019). Stub loaded, low profile UWB antenna with independently controllable notch‐bands. Microw. Opt. Technol. Lett..

[4] Rizvi S.N.R., Awan W.A., Choi D., Hussain N., Park S.G., Kim N. (2023). A compact size antenna for extended UWB with WLAN notch band stub. Appl. Sci..

[5] Hussain Musa, Musa Ali Esraa, Abbas Awan Wahaj, Hussain Niamat, Alibakhshikenari Mohammad, Virdee Bal S., Falcone Francisco (2022). Electronically reconfigurable and conformal triband antenna for wireless communications systems and portable devices. PLoS One.

[6] Awan W.A., Zaidi A., Hussain M., Hussain N., Syed I. (2021). The design of a wideband antenna with notching characteristics for small devices using a genetic algorithm. Mathematics.

[7] Zaidi A., Awan W.A., Hussain N., Baghdad A. (2020). A wide and tri-band flexible antennas with independently controllable notch bands for sub-6-GHz communication system. Radioengineering.

[8] Shome P.P., Khan T., Laskar R.H. (2019). A state‐of‐art review on band‐notch characteristics in UWB antennas. Int. J. RF Microw. Computer-Aided Eng..

[9] Li Yingsong, Li Wenxing, Ye Qiubo (2013). A reconfigurable triple-notch-band antenna integrated with defected microstrip structure band-stop filter for ultra-wideband cognitive radio applications. Int. J. Antenn. Propag..

[10] Devana V.N., Maheswara Rao A., Maheswara Rao A. (2020). A compact 3.1-18.8 GHz triple band notched UWB antenna for mobileUWB applications. IRO Journal on Sustainable Wireless Systems.

[11] Vamseekrishna A., Madhav B.T.P. (2018). A frequency reconfigurable antenna with Bluetooth, Wi-Fi and WLAN notch band characteristics. Int. J. Eng. Technol..

[12] Ahmad S., Ijaz U., Naseer S., Ghaffar A., Qasim M.A., Abrar F., Parchin N.O., See C.H., Abd-Alhameed R. (2022). A jug-shaped CPW-fed ultra-wideband printed monopole antenna for wireless communications networks. Appl. Sci..

[13] Rahman M., NagshvarianJahromi M., Mirjavadi S.S., Hamouda A.M. (2019). Compact UWB band-notched antenna with integrated bluetooth for personal wireless communication and UWB applications. Electronics.

[14] Zhang X., Ur Rahman S., Cao Q., Gil I., khan M.I. (2019). A novel SWB antenna with triple band-notches based on elliptical slot and rectangular split ring resonators. Electronics.

[15] Valizade A., Ghobadi Ch, Nourinia J., Ojaroudi M. (2012). A novel design of reconfigurable slot antenna with switchable band notch and multiresonance functions for UWB applications. IEEE Antenn. Wireless Propag. Lett..

[16] Ghaffar A., Li X.J., Awan W.A., Iffat Naqvi S., Hussain N., Seet B.-C., Alibakhshikenari M., Falcone F., Limiti E. (2021). Design and realization of a frequency reconfigurable multimode antenna for ISM, 5G-sub-6-GHz, and S-band applications. Appl. Sci..

[17] Han Liping, Chen Jing, Zhang Wenmei (2020). Compact UWB monopole antenna with reconfigurable band-notch characteristics. International Journal of Microwave and Wireless Technologies.

[18] Nazeri Amir H., Falahati Abolfazl, Edwards R.M. (2019). A novel compact fractal UWB antenna with triple reconfigurable notch reject bands applications. AEU-International Journal of Electronics and Communications.

[19] Abbas A., Hussain N., Jeong M.-J., Park J., Shin K.S., Kim T., Kim N. (2020). A rectangular notch-band UWB antenna with controllable notched bandwidth and centre frequency. Sensors.

[20] Bong Han‐Ul, Jeong Minjoo, Hussain Niamat, Rhee Seung‐Yeop, Gil Sang‐Keun, Kim Nam (2019). Design of an UWB antenna with two slits for 5G/WLAN‐notched bands. Microw. Opt. Technol. Lett..

[21] Devana VN Koteswara Rao, Maheswara Rao A. (2020). Compact UWB monopole antenna with quadruple band notched characteristics. Int. J. Electron..

[22] Li X.-P., Xu G., Ma M.-R., Duan C.-J. (2021). UWB dual-band-notched lanky-leaf-shaped antenna with loaded half-square-like slots for communication system. Electronics.

[23] Vallappil A.K., Khawaja B.A., Rahim M.K.A., Iqbal M.N., Chattha H.T., Ali M.F.b.M. (2022). A compact triple-band UWB inverted triangular antenna with dual-notch band characteristics using SSRR metamaterial structure for use in next-generation wireless systems. Fractal Fract.

[24] Lin H., Lu Z., Wang Z., Mu W. (2023). A compact UWB monopole antenna with triple band notches. Micromachines.

[25] Iqbal A., Smida A., Mallat N.K., Islam M.T., Kim S. (2019). A compact UWB antenna with independently controllable notch bands. Sensors.

[26] Alnaiemy Yahiea, Nagy Lajos (2021). A novel UWB monopole antenna with reconfigurable band notch characteristics based on PIN diodes. Infocommunications Journal.

[27] Tasouji Nasrin, Nourinia Javad, Ghobadi Changiz, Tofigh Farzad (2013). A novel printed UWB slot antenna with reconfigurable band-notch characteristics. IEEE Antenn. Wireless Propag. Lett..

[28] Mayuri Ponnada, Deepika Rani Nagumalli, Bala Subrahmanyam Nemani, Madhav Boddapati Taraka (2020). Design and analysis of a compact reconfigurable dual band notched UWB antenna. Prog. Electromagn. Res. C.

[29] Trimukhe Mahadu Annarao, Hogade Balaji G. (2019). Compact UWB antenna with tunable band-notch characteristics using varactor diode. Prog. Electromagn. Res. C.

[30] De Arnab, Roy Bappadittya, Kumar Bhattacharjee Anup (2020). Design and investigations on a compact, UWB, monopole antenna with reconfigurable band notches for 5.2/5.8 GHz WLAN and 5.5 GHz Wi‐MAX bands. Int. J. Commun. Syst..

[31] Lakrit Soufian, Das Sudipta, El Alami Ali, Barad Debaprasad, Mohapatra Sraddhanjali (2019). A compact UWB monopole patch antenna with reconfigurable Band-notched characteristics for Wi-MAX and WLAN applications. AEU-International Journal of Electronics and Communications.

[32] Hussain M., Mousa Ali E., Jarchavi S.M.R., Zaidi A., Najam A.I., Alotaibi A.A., Althobaiti A., Ghoneim S.S.M. (2022). Design and characterization of compact broadband antenna and its MIMO configuration for 28 GHz 5G applications. Electronics.

